# Multidisciplinary Understanding of the Urban Heating Problem and Mitigation: A Conceptual Framework for Urban Planning

**DOI:** 10.3390/ijerph191610249

**Published:** 2022-08-18

**Authors:** SangHyeok Lee, Donghyun Kim

**Affiliations:** 1 Marine Policy Research Department, Korea Maritime Institute, 26 Haeyang-ro 301 Beon-gil, Yeongdo-gu, Busan 49111, Korea; 2 Department of Urban Planning and Engineering, Pusan National University, 2 Busandaehak-ro 63 Beon-gil, Geumjeong-gu, Busan 46241, Korea

**Keywords:** urban heating mitigation, heatwave, urban heat island

## Abstract

With the global acceleration of urbanization, temperatures in cities are rising continuously with global climate change, creating an imminent risk of urban heat islands and urban heating. Although much research has attempted to analyze urban heating from various perspectives, a comprehensive approach to urban planning that addresses the problem is just beginning. This study suggests a conceptual framework for multidisciplinary understanding of urban heating by reviewing 147 selected articles from various fields, published between 2007 and 2021, that discuss urban heating mitigation. From these, we identified several outdoor and indoor temperature-reduction factors and proposed area-based, zoning-based, and point-based approaches to mitigate urban heating.

## 1. Introduction

As global warming, rapid urbanization, and industrialization have raised the Earth’s maximum temperature and mean temperature, heatwaves have increased in both intensity and frequency around the world, exceeding adaptive capacity [1,2,3]. A high-alert heatwave is defined as (1) temperatures of or above 35 °C that last at least three consecutive days, or (2) a three-day mean daily temperature of 28 °C or higher with a lowest nightly temperature of 21 °C or higher [4]. The urban heat island effect amplifies the influence of heatwaves on urban areas [5]. Urban heat island (UHI), where urban air temperature is higher than the surrounding rural air temperature, was first observed by Howard [6] (p. 391) and Oke [7], and is cited as a prominent feature of the urban climate [8,9]. UHI can be divided into surface UHI and atmospheric UHI. During the day, buildings and pavements in cities absorb heat and increase surface temperatures by 2 °C, on average, and by 8 °C maximum, contributing to the formation of surface UHI [10]. However, warmer air in urban areas compared to cooler air in nearby rural areas defines atmospheric UHI; the temperature difference becomes evident when a city’s daytime absorbed heat is released at night [11,12]. Urban heating may be understood as the compound effect of global-warming-induced heatwaves and atmospheric UHI.

Cities around the world are making diverse attempts to address urban heating through urban planning, using such approaches as (1) changing land cover or coating, and (2) adjusting the density of urban areas [13]. The former approach attempts to change the cover or coating materials of the city to mitigate urban heating more effectively, using nature-based measures like vegetation cover, green spaces, tree shades, and wind ways, and technology-based measures like replacing heat–susceptible artificial cover and coating materials. The latter approach focuses on the spatial structure of the city and its buildings, mainly discussing the shapes, configuration, and spatial distribution of structures.

Urban heating has long been a subject of studies in various fields, manifesting as discussions about land cover, green spaces, cool pavement, and cool materials. However, the scientific research lacks a systematic integration of previous discussions regarding urban planning [14]. Mitigating urban heating—one of various objectives of human activity in urban areas—becomes a design element in the application of urban planning. There is a common perception that other urban priorities and design elements must be compromised to pursue those that mitigate urban heating; however, these can be integrated to produce plans that more effectively mitigate UHI.

This study reviews the previous research on urban heating reduction in various fields, analyzing the factors relating to urban heating mitigation from the perspective of urban planning. It aims to define a conceptual framework that can be used to formulate new urban planning measures. We used the Web of Science database to collect relevant articles published between 2007 and 2021, using search words like “urban heat island,” “heatwave”, and “urban heat”. In all, 147 articles from various fields were selected and reviewed, including the fields of urban studies, geography, environment, energy, meteorology, built environment and construction, ecology, and climate change (Three articles have been added as per the reviewers’ suggestions. Two articles (#77 and #79) among the 147 do not match Web of Science databases.). Articles about mere meteorological modeling and simulation for climate were excluded. Articles that were case studies and did not include quantitative analyses were excluded. Next, the following four criteria to select articles were applied: those that (1) relate to the real problems of UHI or heatwaves, and not simple simulation; (2) include specific spatial scope in urban space; (3) include interaction of physical factors of urban space and UHI or heatwaves; and (4) discuss urban heating mitigation measures.

This study is not systematic review; rather, it is qualitative theoretical research for a conceptual framework. Of the urban heating mitigation measures that have been studied most intensively, we will first examine those that reduce outdoor temperature. These fall under the categories of buildings and land use, land cover (vegetation, trees, and surface water), and cool pavement. Then, we will consider the urban heating mitigation measures that reduce indoor temperature, which focus on buildings and land use. Lastly, we will integrate these approaches to mitigating urban heating from an urban planning perspective and outline the relevant implications. This study is expected to promote a comprehensive understanding of the urban planning factors that mitigate urban heating.

## 2. Control of Outdoor Temperature

### 2.1. Buildings and Land Use

Density, height, and configuration of buildings are the essential factors in the correlation between UHI and a rise in outdoor temperature. Outdoor temperature increases with less open configuration and a higher density of buildings [15]. Commercial and service areas, which have a higher density of buildings than residential areas, exhibit higher temperatures than residential areas [16]. The heat reflected by high-density structures in urban areas increases UHI by reducing wind speed and changing cloud cover [17,18,19,20]. A space with high-rise buildings exhibits lower surface temperatures than a space with medium- or low-rise buildings because urban canopies create large shadow areas, reducing solar radiation during daytime [21,22]. In addition, the configuration of buildings can increase the reflection of solar radiation or decrease the amount of air circulating around buildings, thereby raising the temperature [23]. The degree of temperature rise may vary depending on the aspect (height/width) ratio and direction of the building; when a building has a lower aspect ratio its surface temperature drops relatively faster, even when exposed to more solar radiation [24,25].

The density, height, and configuration of buildings are linked with the land use in the urban areas where the buildings are located. In urban areas, zoning determines the density and height of buildings. The types of zoning in urban areas influence temperature in association with those of zoning in adjacent areas [26,27,28,29,30]. Commercial zones show higher UHI intensity than do residential zones [27,31]. The effect of UHI is especially high in areas where high-rise buildings are closely situated [9,19]. However, when it comes to the effects of high-rise buildings on temperature rise, opinions are mixed. Some researchers argue that, compared to an open space without vegetation, areas with buildings create shadows during the day, decreasing the heat exposure of pedestrians [21,32]. Others suggest that, in a cluster of buildings with significant height variation, buildings absorb more solar radiation, thus increasing the temperature of the area [33]. High population density in a high-density area also contributes to the rise in temperature [31,34]; in a small, high-density area, poor air circulation can increase nighttime temperatures [35,36].

### 2.2. Land Cover: Vegetation, Trees, and Water

In contrast to urban areas that are covered with impervious layers, vegetation, green spaces, and trees are known as key factors in reducing the outdoor temperature of cities. The expanse of urban areas and consequent diminution of natural areas has changed the surface energy balance, raising both surface and atmospheric temperatures and causing UHI and extreme heat events [37,38,39]. Vegetation cover, green spaces, and trees contribute to the cooling of an area by blocking incoming short-wave radiation, forming shade, allowing potential energy exchange for evapotranspiration [40,41,42], reflecting solar radiation, and reducing the absorption and accumulation of heat [5,15,40,43,44,45,46,47], thereby lowering surface and air temperatures below the level of surrounding city areas. Furthermore, these are considered to be the most effective and economical ways to mitigate the UHI phenomenon [27,39].

Many studies suggest the use of vegetation, green spaces, and trees to change the albedo of the urban surface and mitigate urban heating [48,49,50]. Other options include the following: implementing green roofs, lighter-colored cover, and wide green spaces blocking solar radiation [51]; combining high-albedo roofs and strategic planting around buildings [43,52]; utilizing tree cover to reduce temperature without altering the impervious surfaces of a high-density city [26,53]; and establishing parks and green spaces as open spaces in the city [30,54,55]. In addition to strategies involving vegetation, the literature discusses changing construction materials to absorb solar radiation, combining city parks and urban green infrastructure, and adjusting the proximity of parks to buildings [10,52,56,57,58]. As Table 1 shows, although multiple researchers agree on the positive effects of vegetation, green spaces, and trees on mitigating urban heating, they have different opinions about the degree of these effects.

The effect of land cover (vegetation cover, green spaces, and trees) on UHI mitigation varies depending on the amount, spatial configuration, evapotranspiration, and adaptive capacity of the planting. Although the amount of green space is a key factor in reducing regional temperatures [41,80,81,82], the structure and configuration of green space also influence surface temperatures greatly [76,83,84,85,86,87]. This entails the structure of green space, including its size, shape, and composition [53,76,88,89]; the shape of green space in urban areas [90,91]; and the density of green space in an area [46]. When a green space is 2–16 ha in size, the temperature reduction effect is subject to the configuration of the green space [46] and to the landscape design [92,93]. Particularly, polygonal types of green spaces are more effective in reducing air temperature than are linear green spaces [70,94]. In addition, strategically creating a green space in areas with high heat exposure is more effective at lowering temperatures than increasing the overall green space in the city [3].

Street trees play a significant role in mitigating urban heating. The height of street trees has a negative correlation with surface temperature [95,96], while the cooling effect of tree shading and emission (demonstrated by Lanza and Stone [97]) has a direct influence on pedestrian thermal comfort [5,80,98]. Street trees, used for urban heat mitigation, should be able to maintain a high evapotranspiration capacity and endure heat and drought [99,100]. As water use and evaporation vary depending on tree species, sufficient water supply for the given trees can increase the temperature reduction effect [81,101,102]. Furthermore, it is necessary to consider the direction, height difference, and distance between trees to maximize this effect [72,95,98,103].

The species of street trees, with different evapotranspiration capacities and adaptability, also matter. Isohydric species maintain a constant level of water potential during dry seasons and avoid the damaging influence of drought by lowering stomatal conductance, whereas Anisodydic species decrease water potential and maintain stomatal conductance to survive droughts [104]. Hazel, linden, and maple trees are suggested for dry and hot urban areas [10,99], while Brachychiton discolor, Eucalyptus grandis, and Ficus microcarpa are suggested for Mediterranean climates [101].

In addition to vegetation cover, green space, and trees as land cover measures to reduce urban heating, surface water is also discussed. Water bodies in urban areas, like rivers and lakes, are instrumental in UHI mitigation [19,105,106,107] because they serve as heat sinks [23]. Improving water circulation by intentionally integrating ponds and rivers and incorporating porous surfaces increases surface water potential, thereby mitigating UHI effects [108,109]. Many suggest an increase in surface water to lower the heat load of urban areas [110,111,112,113]. In cities with hot temperatures, including Seoul, Nanjing, and Hiroshima, the potential mitigation effects of vegetation and rivers have been confirmed [114,115].

### 2.3. Cool Pavement in Buildings and Roads

In addition to using vegetation cover, green space, and trees to mitigate urban heating, it is also possible to change the materials used to construct pavement and roofs. A 10% increase in impervious cover in cities raises the regional temperature by 0.7 °C [59]; as such, pavement is a key factor in reducing heat in urban areas. To more effectively mitigate the influence of pavement on UHI, we should reduce the amount of heat emitted from the surface of pavement [116,117]. In other words, we must use cool materials with high solar reflectance and high spectral radiance to increase albedo and reduce temperature [42,118,119]. Cool materials are cost-efficient, reduce energy demand, and improve the urban microclimate [120,121,122]. These can be applied to produce cool pavement and cool roofs, as well as other construction materials [123,124].

Cool roofs, or reflective roofs, have been designed to lower the temperature of the roof surface, reducing the heat flux in the atmosphere [125]. The reflective covering of cool roofs uses highly reflective white coatings, infrared reflecting pigments, and a reflective type of paint to increase albedos; this decreases surface temperature and sensible heat flux in the atmosphere, lowering urban heat by 1.2–2.0 K and surface heat by a maximum of 20 K [52,117]. The relative rise of emissivity plays a crucial role in mitigating urban heating when the reflectivity of the cool materials falls. When cool materials increase roof albedo from 0.3 to 0.7, summertime temperature decreases by 9.6 °C in high-density areas and by 11.3 °C in residential areas [126,127]. Changing to a cool roof results in a surface temperature decrease that is approximately 44% lower than when using concrete material [128].

In addition to roofs, cool materials can be applied to the surface of streets and roads as cool pavement. An increase of 0.25 in the albedo of pavement material translates into a reduction of 6.8 K in surface temperature, and an increase of 0.6 in albedo entails a reduction of 20 K [129]. Furthermore, cool pavement reduces temperatures of surrounding areas by up to 2.1 K [130], and by up to 1.6 K during summertime [131]. Pavement using cool materials changes the concrete itself by making it self-cooling. In contrast to conventional concrete pavement, self-cooling concrete pavement lowers surface temperature by 7K [132]. Previous concrete pavements proved to have a cooling effect through the re-evaporation of water [133].

The use of cool materials may be effective in hot and arid climates; however, their use in cold and humid continental climates must be carefully considered, given that they may decrease the benefits of heat [134,135,136]. Even in a hot and arid climate, an increase in solar reflection due to cool pavement in urban areas can increase the heat stress of pedestrians [137,138]. It is cost-efficient to use cool materials; however, overall, it is more effective to rely on vegetation cover, green space, and trees to mitigate UHI effect [22,122,128]. The most effective means of lowering UHI intensity is reducing building density and paved surface area while simultaneously increasing green space and surface water in high-density areas [111,128].

## 3. Control of Indoor Temperature

Outdoor temperature and human activities determine indoor temperature [15,139]. In discussions on indoor temperature, thermal comfort is regarded as an important factor in protecting the residents of a building against extreme weather conditions, including heatwaves [140]. Thermal comfort inside buildings is considered neutral at a temperature of 26 °C, and overheating at 28 °C or above [141]. In urban areas, the indoor temperature tends to be 3 °C higher than that of surrounding rural areas due to the UHI effect [142]. Although UHI reduces heating energy demands during the winter as a trade-off for increased cooling energy demands during the summer, the losses far outweigh the benefits in terms of greenhouse gas emission, energy expenses, and human health [11,143,144]. Elements that may lower indoor temperature to maintain thermal comfort and prevent overheating of buildings include building design, construction materials, and land use.

Building design and construction materials are discussed in association with energy consumption to maintain thermal comfort. The thermal performance of housing is a key factor influencing indoor temperature: buildings’ thermal resistance helps to enhance the heat adaptability and health of urban residents [145,146]. Cooling energy consumption increases as the height of a building and the mean height of the surrounding buildings increase [12,24,146], and solar radiation increases the energy demands of buildings facing south and east [24,147]. Regarding construction materials, the use of light building materials increases cooling energy consumption, and the cooling effect is better when using external sun protection than when using internal sun protection [141,143,144,148].

To lower indoor temperature and cooling energy demand, the following shading options can be selected: windows (double pane window, and blinds), walls (cool facade and green facade), and roofs (cool roof and green roof) [4,142,144,145,148,149,150,151]. In addition, reflective materials [119,136,146,150,152,153] and vegetation [10,57,119,146] can be applied to the surface of buildings, including roofs and walls. The advantage of reflective materials is the ease of design and maintenance [152]. The lighter (or whiter) the color of the surface, the higher the albedo [120,136,154]. However, the effects of temperature reduction measures vary depending on differences in solar radiation, which arise due to the direction of buildings [12,24,151].

As with controlling outdoor temperature, land use is another way to reduce indoor temperature. Trees, green spaces, and surface water in and around buildings lower surrounding temperatures, contributing directly and indirectly to reduced energy consumption in buildings [5,10,43,57,139,145,149,154,155,156]. In addition, due to the UHI effect, the use of buildings in a specific zone may influence energy demand. For example, commercial and service buildings show higher energy consumption than residential buildings [16,157,158]. The discussion of land use is often related to the heat vulnerability of the vulnerable class of a city. Heatwaves and UHI greatly affect elderly people and those with potential heat-related illnesses [142,159,160]. The provision of easy-to-access shaded areas, water bodies, and green spaces with cooler temperatures than those indoors is necessary to help the heat-vulnerable population [159]. Particularly, due to the ability of green space to purify air pollutants as well as reduce indoor and outdoor temperatures, green space is discussed as a crucial factor in considering land use [161].

## 4. Discussions

Urban heating is one of the most important issues in urban planning, and it is caused by two unavoidable global trends: climate change and urbanization. The plethora of problems caused by urban heating include heat-related illnesses, public health risks, increased energy consumption, air pollution, and labor productivity loss [14,37,162,163]. Despite all the studies on urban heating in diverse areas, the discussion of a comprehensive framework that can be applied to urban planning is still in its early stage [2]. It is only recently that a few studies have approached the issue of urban heating from the perspective of urban planning.

Stone Jr. et al. [13] constructed a combined strategic scenario—a greening scenario, cool material scenario, and energy efficiency scenario—and linked it with the assessment of heat-related mortality. Wheeler et al. [2] approached the issue with three greenspace strategies in their framework on built form. They integrated several previously suggested mitigation measures regarding land cover and buildings into scenarios, then evaluated these using public health criteria. Although their approach is useful because it focuses on people, it cannot be integrated with a developmental scenario due to its focus on adaptation planning.

To overcome the limitations of Stone Jr. et al.’s [13] scenario, Heris et al. [164] suggested a framework wherein they approach microclimate management as development management. They found five main factors shaping the policy choices taken to mitigate urban heating: (1) urban vision, (2) land use and form controls, (3) design guidelines, (4) public financing, and (5) ownership/condemnation. These factors, which emphasize the planning process more so than detailed technical measures, are meaningful insofar as they make urban heating mitigation in urban development mainstream. They cover stakeholders’ understanding of urban heating mitigation, regulatory and design guidelines to achieve policy goals, financing, and ownership conflicts in the implementation process. This approach assumes public intervention through regulatory policies, which unavoidably limits the elements and actions that can be used in implementation.

Mahlkow and Donner [14] raised awareness of the barriers to implementing adaptation plans and policies regarding urban heating. They conducted a constellation analysis on heat stress using “StEP Klima,” a policy instrument that considers stakeholders’ needs, and divided symbolic elements—policy, initiative, strategy, plan, technical elements, and natural elements—to suggest a framework where the elements are linked with the actors. The strength of this framework is in its organization and implementation of plans: it creates feasible action plans by engaging actors and separating the elements of action. However, its planning process (to address urban heating) is vague and insufficient to organize urban development plans that integrate heat mitigation measures from a spatial perspective.

We would like to recommend two avenues for future research based on the comprehensive framework of this study. First, we propose a study to derive a planning alternative that integrates various measures into a comprehensive framework at the city level and to quantitatively estimate its effect. The comprehensive framework proposed in this study includes measures to mitigate heatwaves for various planning units of cities, such as area-based, zoning-based, and point-based approaches. The effect of each individual measure is supported by previous quantitative studies; however, the combined or integrated effect at the city level is unknown. When the effects of each measure are integrated at the city level, synergies and trade-offs among various measures may appear. The simulation of the integrated effect on measures at the city level could fill the gap in existing heatwave-related studies. Second, we recommend a plan evaluation study regarding which measures are being used for heatwave mitigation in the urban comprehensive plan. In order to effectively mitigate heatwaves at the city level, various measures from among the three approaches proposed in this study should be considered. Understanding which approaches and measures are suitable for each city will contribute to identifying the gaps in composing urban plans for heat wave reduction in the future.

## 5. Conclusions

**Figure 1:**
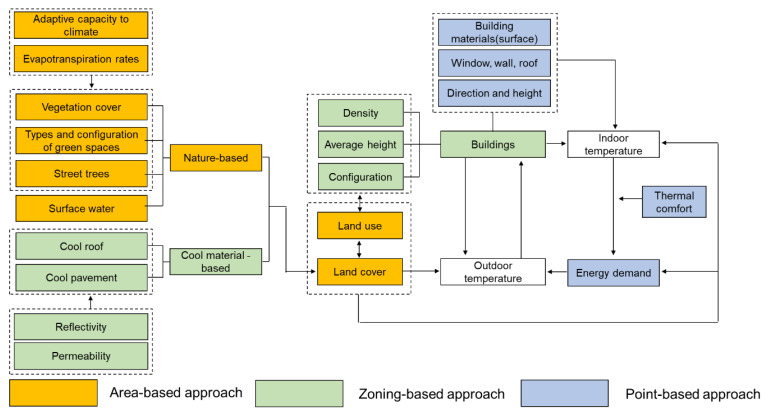
Comprehensive framework for understanding urban heating in urban planning.

As Figure 1 shows, we established a comprehensive framework applicable to urban planning based on the review of studies on urban heating in a wide range of areas. To address urban heating from the perspective of urban planning, we should aim to control outdoor temperature—consisting of surface and air temperature—and indoor temperature, which influences human health. Land cover and buildings are key factors influencing outdoor temperature, and they are associated with land use in urban planning. Building design, including materials and outdoor temperature, mainly influences indoor temperature. In other words, the type of land use determines the type of regulations applied to the building. Thus, temperature reduction should be integrated into urban planning focused on land use to mitigate urban heating.

We would like to suggest the following three approaches to urban planning to mitigate urban heating based on land use: area-based, zoning-based, and point-based. The area-based approach is a strategy for organizing land cover and land use from the perspective of urban spatial structure. As urban heating mitigation is based on land cover, this approach tries to determine what spatial structure vegetation cover should assume in urban areas, and how this vegetation cover should be integrated with the land use in urban areas. In addition, this approach deals with the amount and structure of vegetation cover—a nature-based solution—and how a network of vegetation cover can be created in an urban area. The zoning-based approach is a strategy for mid- and-high density development districts, where lack of vegetation cover is common. This approach can serve as a guideline for density, height, and configuration of buildings based on the type of land use. In addition, it is a strategy to find cool materials-based solutions for mid- and-high density areas, whose problems cannot be solved with vegetation-based solutions alone. As density and thermal environment differ according to different types of land use, regulations are applied in association with zoning. The point-based approach is a strategy for individual buildings that reduces indoor temperature through design guidelines and energy efficiency measures. This approach provides response measures regarding construction materials, windows, walls, roofs, and the direction and height of buildings. Notably, this approach addresses policies pertaining to individual buildings, which need to implement temperature reduction measures to minimize the negative effects of urban heating for heat-vulnerable groups of people.

## Figures and Tables

**Table 1:** Effects of vegetation, green spaces, and trees on mitigating urban heating.

Research	Main Results	Area
Klok et al. [59]	Green space accounts for 69% of temperature change.	Rotterdam
Guo et al. [60]	Normalized Difference Vegetation Index (NDVI) and Land Surface Temperature (LST) show a strong negative correlation.	Guangzhou
Heusinkveld et al. [8]	Green space reduces temperature by 4.0 K in urban areas.	Rotterdam
Wang et al. [22]	A 10% increase in vegetation cover in urban areas lowers mean air temperature by 0.5–0.8 °C.	Toronto
Peron et al. [10]	Replacing existing cover with cool materials and pervious green spaces lowers temperature by 4 °C.	Venice
Li et al. [61]	A 10% increase in green space lowers LST by 0.86 °C.	Beijing
Sung [62]	There is a strong negative correlation between the ratio of tree-covered area and mean surface temperature.	Woodland (Texas)
Chow et al. [63]	Green space lowers temperature by 1–3 °C, and sometimes by up to 5 °C.	Phoenix
Klemm et al. [64]	A 10% increase in tree planting lowers mean temperature in urban canyons by 1 K.	Utrecht
Armson et al. [65]	Green space reduces surface temperature in urban areas by 20 °C maximum, and tree shade lowers overall temperature by 5–7 °C.	Manchester
Coutts and Harris [66]	A 10% increase in vegetation cover lowers surface temperature by approximately 1 °C.	Melbourne
Qiu et al. [27]	Green space in urban areas mitigates UHI intensity by 1.57 °C.	Shenzhen
Zölch et al. [3]	Maximum saturation of tree planting can lower Physiological Equivalent Temperature (PET) by up to 10–13%.	Munich
Middel et al. [67]	A 25% increase in urban tree canopy cover lowers air temperature by 2 °C.	Phoenix
Sun and Chen [68]	The effect of green spaces on surface temperature is 1.64 °C for forests and 2.21 °C for lawns.	Beijing
Zhang, Murray et al. [69]	Increasing green spaces lowers LST by approximately 1–2 °C locally, and by 0.5 °C regionally.	Phoenix
Park et al. [70]	Polygonal and mixed type small green spaces lower temperature by 1 °C per 300 m^2^ area and 2300 m^3^ volume, and 2 °C per 650 m^2^ area and 5000 m^3^ volume.	Seoul
Ng et al. [71]	Lowering the temperature of an urban area by 1 °C requires tree planting in 33% of that area.	Hong Kong
Tan et al. [72]	In contrast to exposed surfaces, tree shade on the road lowers air temperature by 15.9–18.8 °C, and 1–1.5 °C at the pedestrian level.	Hong Kong
Hamada and Ohta [73]	The maximum temperature difference between urban areas and green spaces is 1.9 °C.	Nagoya
Lee et al. [74]	The mean temperature difference between urban areas and green spaces is 1.76 °C.	Seoul
Wang and Shu [39]	A 10–20% increase in vegetation cover is anticipated to reduce UHI by 0.38–0.78 °C.	Shanghai
Dutta et al. [30]	Green park has a cooling effect of 0.938 °C up to 50 m from the boundary, and 0.283 °C lower on average at 50–100 m.	India
Dialesandro et al. [53]	Urban forest landscape cools daytime temperatures by 5.6 °C compared to the metropolitan average.	Dryland urban regions (Cairo, Delhi, etc.)
Gao et al. [52]	The maximum cooling effect of green roof is 1–1.2 K.	Xi’an and Wuhan
Žuvela-Aloise et al. [75]	0.5 °C can be reduced if all roofs in the entire area are changed to green roofs.	Vienna
Shah et al. [76]	The average temperature difference between urban areas and green spaces is 2.23 °C.	Bengaluru (India)
Bianchi et al. [77]	The UHI effect in urban areas is due to hot exhaust gas from traffic, asphalt, and concentration of soot (no vegetation area).	Salt Lak Valley (Utah)
Zinzi and Carnielo [78]	The UHI effect ranges between 0.7 and 1.8 °C and a maximum of 8.1 °C hourly. The large vegetation areas take advantage of evaporative cooling.	Rome
Street et al. [79]	The energy use intensity of urban areas (little vegetation) is higher than that of rural areas (13% for a small office, 17% for a single family).	Boston

Note: We added three articles (#77–#79) during the review process. Article #77 is not included in the index of Web of Science, and article #79 is a conference article.
